# Additively Manufactured Carbon-Reinforced ABS Honeycomb Composite Structures and Property Prediction by Machine Learning

**DOI:** 10.3390/molecules29122736

**Published:** 2024-06-08

**Authors:** Meelad Ranaiefar, Mrityunjay Singh, Michael C. Halbig

**Affiliations:** 1 NASA Glenn Research Center, Cleveland, OH 44135, USA; michael.c.halbig@nasa.gov; 2 Ohio Aerospace Institute, Cleveland, OH 44142, USA

**Keywords:** acrylonitrile butadiene styrene (ABS), additive manufacturing, carbon-reinforced ABS, classification, fused filament fabrication, honeycomb, machine learning, mechanical properties, polymer composites, regression

## Abstract

The expansive utility of polymeric 3D-printing technologies and demand for high- performance lightweight structures has prompted the emergence of various carbon-reinforced polymer composite filaments. However, detailed characterization of the processing–microstructure–property relationships of these materials is still required to realize their full potential. In this study, acrylonitrile butadiene styrene (ABS) and two carbon-reinforced ABS variants, with either carbon nanotubes (CNT) or 5 wt.% chopped carbon fiber (CF), were designed in a bio-inspired honeycomb geometry. These structures were manufactured by fused filament fabrication (FFF) and investigated across a range of layer thicknesses and hexagonal (hex) sizes. Microscopy of material cross-sections was conducted to evaluate the relationship between print parameters and porosity. Analyses determined a trend of reduced porosity with lower print-layer heights and hex sizes compared to larger print-layer heights and hex sizes. Mechanical properties were evaluated through compression testing, with ABS specimens achieving higher compressive yield strength, while CNT-ABS achieved higher ultimate compressive strength due to the reduction in porosity and subsequent strengthening. A trend of decreasing strength with increasing hex size across all materials was supported by the negative correlation between porosity and increasing print-layer height and hex size. We elucidated the potential of honeycomb ABS, CNT-ABS, and ABS-5wt.% CF polymer composites for novel 3D-printed structures. These studies were supported by the development of a predictive classification and regression supervised machine learning model with 0.92 accuracy and a 0.96 coefficient of determination to help inform and guide design for targeted performance.

## 1. Introduction

The continued advancement of tools and technologies to meet lightweight and high-performance needs demands the development and utilization of novel multi-functional composite structures. To this end, additive manufacturing (AM), commonly referred to as 3D-printing, represents a significant advancement in conventional manufacturing by enabling the fabrication of complex, tailored components and offering compatibility with a broad spectrum of materials. AM further deviates from traditional subtractive approaches and is instead characterized by the layer-by-layer assembly of intricate geometries directly from computer-aided design models. This has led to novel applications in the automotive, aerospace, maritime, energy, and medical sectors through the development of components, including crankshafts, heat exchangers, batteries, and dental implants [1,2,3,4].

One notable technique under the umbrella of AM is fused filament fabrication (FFF), also referred to as fused deposition modeling (FDM); it has been made popular due to its versatility, cost-effectiveness, and low barrier to entry [5]. In this process, a spool of filament material is fed through a heated nozzle and deposited onto a substrate layer-by-layer to construct bespoke three-dimensional objects. Depending on the material, such as high-performance thermoplastics and ceramics, a heated substrate and heated chamber could be necessary to address print defects, improve layer-to-layer adhesion, and enhance print quality [6]. The properties and fabrication of components from polylactic acid filament with metal-reinforcement for added multi-functionality, carbon-reinforced PEEK and PEI filament, and sustainable naturally reinforced filament with wood and hemp have also been explored [7,8,9]. Some FFF printers also allow the user to incorporate continuous-fiber reinforcement, such as Kevlar for added impact resistance [10]. The utilization of multiple nozzles on a single FFF printer further enables the fabrication of tailored multi-material and multi-functional components through the use of multiple filaments in a single print task [11]. A secondary nozzle can also be leveraged to incorporate sacrificial filament within a design, supporting overhangs and other typically difficult-to-print geometries; this sacrificial filament is later removed through dissolution.

Taking full advantage of the design freedom and tailorability afforded by FFF, opportunities to advance technology through topology optimization, integrated features, and part consolidation for reduced weight and improved performance will play a critical role in FFF’s adoption by industry [12,13]. One specific avenue to address lightweighting through FFF of components is by incorporating lattice design, which can be achieved through the use of triangular, grid, rectilinear, and honeycomb infill patterns [14]. The honeycomb pattern, a hexagonal (hex) arrangement of cells, is a prevalent architecture in nature that is most recognized for its role in beehive structures; it provides a high strength-to-weight ratio and packing density and is currently used by the aerospace industry for the design of lightweight aluminum aircraft panels. In a study by Mansour et al. [15], an examination of the honeycomb structure and the reinforcement material determined that incorporating a hierarchical structure with chopped fiber or carbon nanotubes results in increased strength and stiffness. In this light, FFF opens the door for the development of complex multi-material and multi-functional honeycomb structures with great potential for application in the aerospace industry.

Addressing the need for both novel lightweight structures and an increased desire for sustainable closed-loop systems toward a circular economy, acrylonitrile butadiene styrene (ABS) and polylactic acid (PLA) are widely available material systems that are compatible with low-cost FFF printers and have demonstrated qualities of biodegradability and recyclability [16,17,18]. Relative to PLA, ABS offers better impact, heat, and electrical resistance properties and has been widely investigated in order to determine the effects of processing parameters and reinforcement materials on mechanical properties, tribological behavior, and thermal properties [19,20,21,22]. However, the numerous features involved in 3D-printing create a large and complex design space, which has not yet been fully explored and requires additional investigation. With sufficient development and understanding of the process–structure–property relationships of the ABS system along with the effect of material reinforcement, FFF components with tailored designs could be developed to meet performance needs. By leveraging machine learning and artificial intelligence approaches, these relationships and discoveries could then be utilized to accelerate, inform, and guide designs for targeted properties [23,24,25,26,27].

In the current work, ABS and reinforced ABS materials were printed by desktop FFF. Process parameters, including print-layer height and hex size, were modified to ascertain their effects on the resultant 3D-printed structures and properties. This was achieved through the characterization and evaluation of specimens by microscopy and compression testing. Furthermore, machine learning models trained on experimental results were developed to classify materials and predict ultimate compressive strength; these were subsequently evaluated for performance and can provide a means to guide and inform the design of these honeycomb structures.

## 2. Results and Discussion

### 2.1. Material Characterization

**Figure 1:**
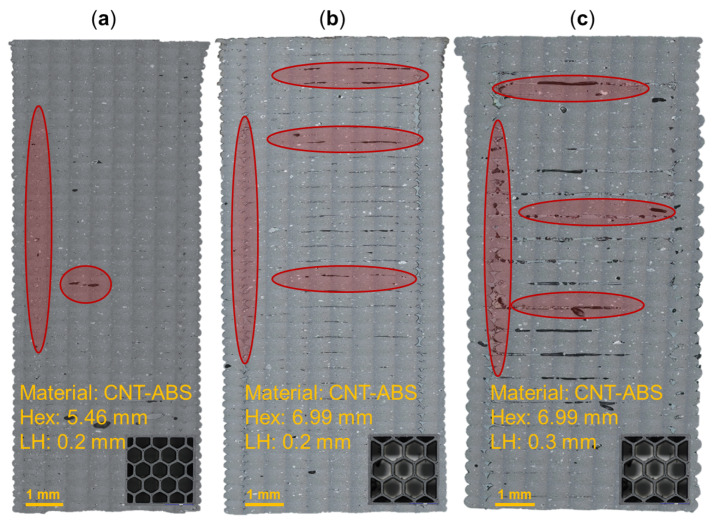
Optical microscopy cross-sections, in-plane with the print direction, of carbon nanotube reinforced (CNT) ABS specimens with different hex sizes and print-layer heights (LHs): (**a**) Hex: 5.46 mm|LH: 0.2 mm; (**b**) Hex: 6.99 mm|LH: 0.2 mm; (**c**) Hex: 6.99 mm|LH: 0.3 mm. A selection of porosity defects is highlighted in red. A cross-section perpendicular to the print direction corresponding to the 5.46 mm and 6.99 mm hex sizes and each having a length and width of approximately 25.4 mm × 25.4 mm is also provided for each specimen.

The structures of 3D-printed components, including defects such as porosity, are influenced by the combination of process parameters used during fabrication. Hex sizes ranging from 5.46 mm to 6.99 mm and print-layer heights of 0.2 mm and 0.3 mm were investigated for ABS, CNT-ABS, and CF-ABS materials (3DXTech, Grand Rapids, MI, USA). Figure 1 illustrates the effects of the hex size and print-layer height (LH) on FFF CNT-ABS for several process combinations. It is observed in Figure 1a that a cross-section of CNT-ABS having the smallest hex size of 5.46 mm and shortest layer height of 0.2 mm contains relatively few defects, which are highlighted in red. When increasing the hex size to the maximum-printed 6.99 mm while maintaining the layer height at 0.2 mm, as shown in Figure 1b, there is an increase in inter-layer porosity found throughout the cross-section. Maintaining the larger hex size of 6.99 mm and increasing the print-layer height to 0.3 mm is then shown to significantly increase inter-layer porosity relative to the other process parameter combinations. When maintaining all other parameters as constant, a smaller print-layer height improves packing, overlap, and adhesion [28,29]. For these reasons, relative to a 0.2 mm print-layer height, the 0.3 mm print-layer height had increased air gaps and porosity. The increase in porosity with increasing hex size could potentially stem from changes in the print duration. As the hex size increases, an individual layer’s surface area decreases, and this corresponds to a decrease in print time. The resulting thermal gradient and its effect on layer adhesion could influence porosity formation. However, a comprehensive analysis of porosity at different hex sizes but at a constant print-layer height is necessary to further investigate this relationship. Additionally, across all process parameter combinations, there is porosity observed along the full length of the honeycomb vertices. The consistency of this porosity in each cross-section can then be explained by the printer’s nozzle path during fabrication and possible lack of overlap or under-extrusion of filament to fully connect the honeycomb walls at the vertices. A modification to the print process parameters, such as by increasing the overlap percentage, would likely resolve this defect.

**Figure 2:**
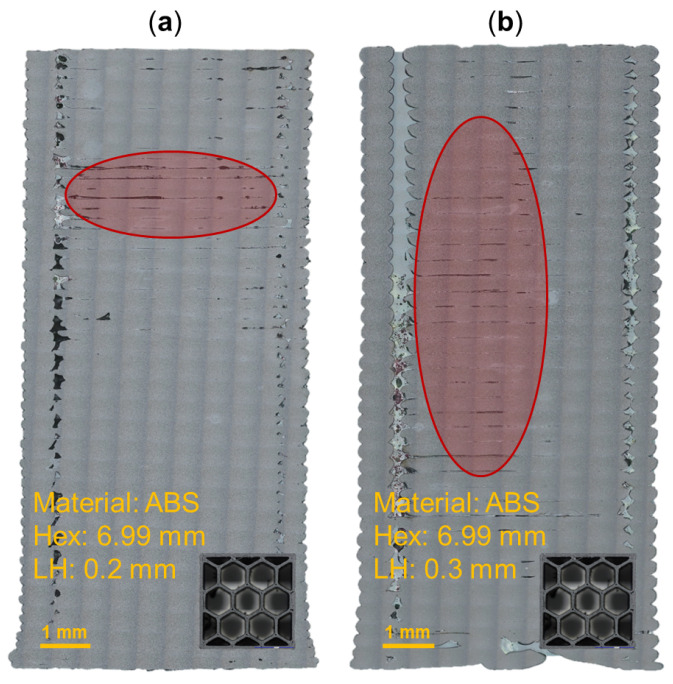
Optical microscopy cross-sections, in-plane with the print direction, of pure ABS specimens with a constant hex size and different layer heights (LHs): (**a**) Hex: 6.99 mm|LH: 0.2 mm; (**b**) Hex: 6.99 mm|LH: 0.3 mm. A selection of porosity defects are highlighted in red. A cross-section perpendicular to the print direction corresponding to the 6.99 mm hex size and each having a length and width of approximately 25.4 mm × 25.4 mm is also provided for each specimen.

With this perspective, the pure ABS honeycomb cross-sections in Figure 2 illustrate the effect of layer height on porosity for the largest printed hex size. A print-layer height of 0.2 mm with a hex size of 6.99 mm (Figure 2a) demonstrates partial inter-layer porosity that is mostly contained in the upper half of the print. When the print-layer height is increased to 0.3 mm and the hex size remains constant at 6.99 mm (Figure 2b), the observed inter-layer porosity increases: extending through a larger portion of the cross-section. These ABS honeycomb specimens demonstrate an increase in porosity with increasing print-layer height, in agreement with trends founds in the CNT-ABS honeycomb specimens. However, it is also observed that the inter-layer porosity found in ABS honeycomb cross-sections is not as significant relative to CNT-ABS specimens fabricated under the same hex size and print layer height conditions. This is possibly due to a combination of process parameters and an increased propensity for micro-porosity in CNT-ABS, which are demonstrated by the speckled features throughout the cross-sections in Figure 1. The result is weaker interfaces and more extensive porosity overall compared to printing with pure ABS. In this context, a general solution to improve the print quality is to use a smaller print-layer height at the trade-off of the time savings achieved through increased print-layer heights. Additionally, similar to the CNT-ABS specimens, the ABS cross-sections demonstrate inherent porosity along the honeycomb vertices due to the nozzle path and under-extrusion. However, the increased number of discontinuous regions in Figure 2 relative to Figure 1 is a result of the chosen process and print parameters. Given constant processing conditions such as nozzle diameter, nozzle speed, and print bed temperature, the resulting print quality can vary based on adjustments to the hex size, print-layer height, and material. For the same processing parameters, the increased thermal conductivity of CNT-ABS relative to ABS could result in increased material extrusion and overlap as well as reduced discontinuities in the print. In both materials, these discontinuities could be remedied by modifying process parameters to provide additional material extrusion for connecting the honeycomb vertices. For additional microstructure analysis and porosity quantification, the reader is referred to prior work [30].

**Figure 3:**
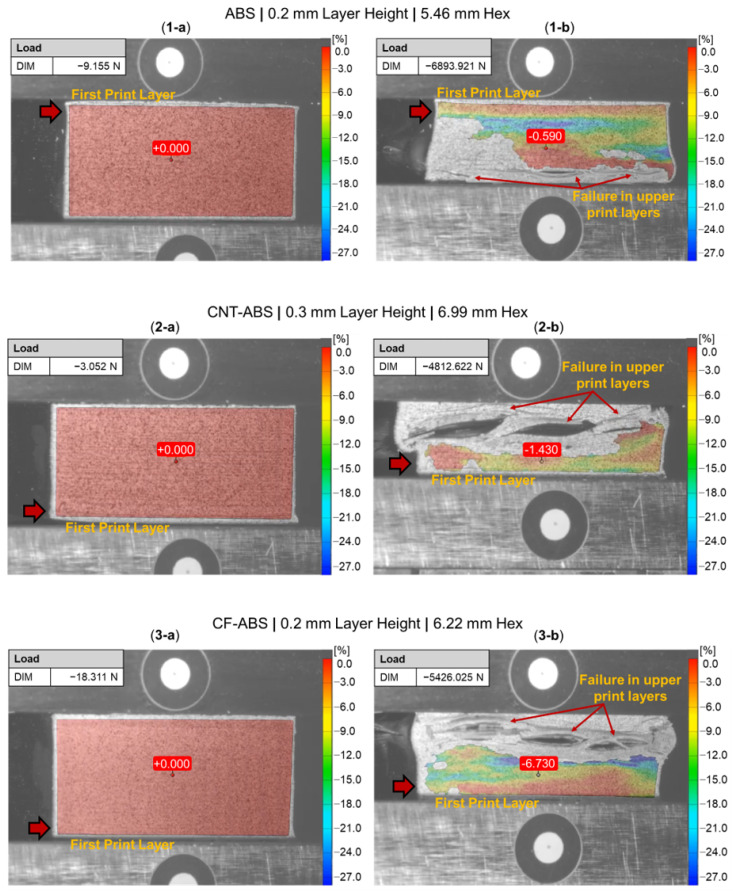
DIC snapshots of honeycomb specimens during compression testing: (**1**) ABS with 5.46 mm hex size and 0.2 mm layer height; (**2**) CNT-ABS with 6.99 mm hex size and 0.3 mm layer height; (**3**) CF-ABS with 6.22 mm hex size and 0.2 mm layer height; (**a**) minimal applied load; (**b**) after yielding. The first print layer and the location of failure after yielding are noted.

Figure 3 provides a digital image correlation (DIC) snapshot of several honeycomb specimens during compression testing at a minimal applied load and after yielding. It is noted that the orientation of coupons within the test frame was varied such that the coupon was resting on either its first or last print layer. The 5.46 mm hex and 0.2 mm print-layer height ABS honeycomb specimen (Figure 3(1-a,1-b)) was tested while resting on its last print layer. Upon compressive yielding, the bottom of the specimen, corresponding to the last print layers, begins to demonstrate inter-layer delamination. Examination of the CNT-ABS specimen with a 6.99 mm hex and 0.3 mm print-layer height in Figure 3(2-a,2-b) revealed a similar location of failure in the region corresponding to the upper half of the print. Furthermore, failure in the upper print layers also occurred with the 6.22 mm hex and 0.2 mm print-layer-height CF-ABS honeycomb specimen in Figure 3(3-a,3-b). The proximity of the failure to the upper print layers of these honeycomb specimens was identified as a consistent trend across all tested honeycomb specimen variations, regardless of specimen orientation in the test frame. This is likely an artifact of the FFF process, processing parameters, and material. When printing the first layers of a specimen, the build plate provides an additional heat source to help reduce warping and improve adhesion between each print layer. As the print extends further away from the build plate, the effect of the heated build plate is diminished, resulting in reduced inter-layer adhesion and print quality [31,32]. This is supported by imaged cross-sections in Figure 1 and Figure 2, where a gradual increase in porosity in the latter half of print layers can be observed. A potential solution to improve layer adhesion throughout the print is to leverage a fully enclosed chamber and a preheating period to bring the internal chamber environment to a sufficiently high and uniform temperature. As a result of increased porosity in the upper print layers, this region of the specimen is inherently weaker and susceptible to becoming the initial location of failure upon yielding. Additionally, it is observed that compressive failure in the carbon-reinforced ABS is generally more destructive than that of the pure ABS honeycomb specimens. This could, again, be explained by increased porosity in the carbon-reinforced specimen relative to pure ABS specimen, resulting in the more extreme inter-layer delamination after yielding observed in Figure 3(2-b,3-b).

### 2.2. Mechanical Properties

**Figure 4:**
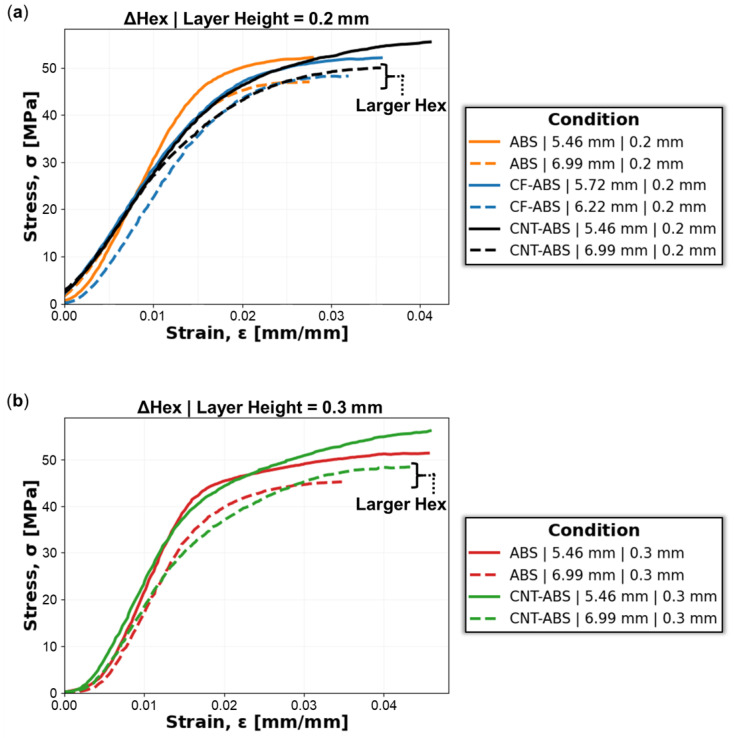
Stress–strain curves for the compression testing of 3D-printed honeycomb coupons: (**a**) ABS and CNT-ABS specimens with 5.46 mm and 6.99 mm hex sizes as well as CF-ABS specimens with 5.72 mm and 6.22 mm hex sizes, where all specimens have a constant print-layer height of 0.2 mm; (**b**) ABS and CNT-ABS specimens with 5.46 mm and 6.99 mm hex sizes, where all specimens have a constant print-layer height of 0.3 mm. Solid lines represent the smaller hex size (5.46 mm or 5.72 mm), and dashed lines represent the larger hex size (6.2 mm or 6.99 mm) of each material.

Figure 4 shows a representative subset of data obtained from compression testing of ABS, CF-ABS, and CNT-ABS honeycomb specimens with hex sizes of 5.46 mm, 5.72 mm, 6.22 mm, and 6.99 mm for print-layer heights of 0.2 mm and 0.3 mm. When maintaining a constant print-layer height while varying the material and hex size, it is observed in Figure 4a that the ABS, CF-ABS, and CNT-ABS specimens with hex sizes of 5.46 mm, 5.72 mm, and 5.46 mm, respectively, withstood larger stresses than their larger-hex-sized counterparts of 6.99 mm, 6.22 mm, and 6.99 mm, respectively. The ABS and CNT-ABS specimens with a print-layer height of 0.3 mm, shown in Figure 4b, demonstrated similar behavior, with the smaller 5.46 mm-hex-size specimens undergoing larger stresses before failure relative to the 6.99 mm hex specimens. These observations reinforce a trend of decreasing compressive strength with increasing hex size and are further corroborated by forthcoming results and analysis.

**Figure 5:**
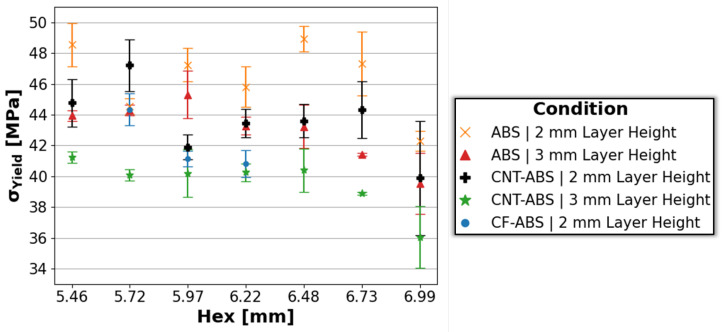
Average compressive yield strengths with standard deviation error bars for ABS, CNT-ABS, and CF-ABS across all hex sizes (5.46 mm, 5.72 mm, 5.97 mm, 6.22 mm, 6.48 mm, 6.73 mm, and 6.99 mm) and print-layer heights (0.2 mm and 0.3 mm).

Figure 5 shows the average compressive yield strength, $\sigma_{yield}$, with standard deviation error bars for ABS, CNT-ABS, and CF-ABS across all hex sizes, where the compressive yield stress for each specimen is determined from the 0.2% offset plastic strain. Observing the overall trend of the data, there is a slight negative correlation of decreasing compressive yield strength with increasing hex size, in agreement with trends from Figure 4. Additionally, the yield strengths for ABS and CNT-ABS with 0.2 mm print-layer height are consistently greater than their counterparts with 0.3 mm print-layer height. This is due to increased inter-layer porosity with increased print-layer heights, as observed in Figure 1 and Figure 2, resulting in reduced strength for the 0.3 mm print-layer-height specimens relative to the 0.2 mm print-layer-height specimens. When comparing ABS and CNT-ABS, it is shown that ABS generally achieved higher compressive yield strength across all hex sizes. For the case of CF-ABS with 0.2 mm print-layer height, the determined yield strength was lower than the values for all other feature combinations except CNT-ABS with 0.3 mm layer height. The performance of CF-ABS specimens falling between that of the CNT-ABS 0.2 mm and 0.3 mm print-layer height specimens and below that of ABS honeycombs is an expected outcome based on the increased porosity observed in the carbon-reinforced specimens. CF-ABS with 0.2 mm print-layer height likely demonstrated reduced compressive yield strength relative to CNT-ABS with 0.2 mm print-layer height due to an increased quantity of porosity within the filament and, consequently, increased porosity within the printed component, as described by Vakharia et al. [7]. If CF-ABS with 0.3 mm print-layer height had been 3D-printed, it would likely have the lowest compressive strength of all tested feature combinations.

**Figure 6:**
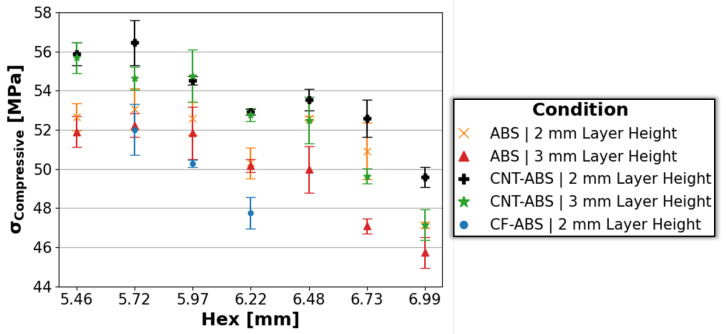
Average ultimate compressive strength with standard deviation error bars for ABS, CNT-ABS, and CF-ABS across all hex sizes (5.46 mm, 5.72 mm, 5.97 mm, 6.22 mm, 6.48 mm, 6.73 mm, and 6.99 mm) and print-layer heights (0.2 mm and 0.3 mm).

The average ultimate compressive strength, $\sigma_{compressive}$, and standard deviation error bars, determined by the maximum stress, for ABS, CNT-ABS, and CF-ABS specimens across all hex and layer heights is captured in Figure 6. A strong negative correlation is readily observed in this instance, where the ultimate compressive strength decreases with increasing hex size, from 5.46 mm to 6.99 mm, for 0.2 mm and 0.3 mm print-layer heights. For each ABS and CNT-ABS, it is observed that the 0.2 mm print-layer height resulted in a larger ultimate strength than the 0.3 mm print-layer height, resulting from increased porosity with increasing layer height. However, unlike with compressive yield strength, the CNT-ABS specimens of both layer heights achieved larger ultimate compressive strength values than ABS honeycombs across all hex sizes. This is likely due to the initial compressive load compacting the inter-layer porosity present in coupons combined with enhanced CNT reinforcement, resulting in substantial strengthening of CNT-ABS specimens before failure. Producing the lowest ultimate strength values, CF-ABS specimens of 0.2 mm print-layer height and 5.72 mm, 5.97 mm, and 6.2 mm hex sizes likely suffered from increased porosity, contributing to an overall weaker performance relative to ABS and CNT-ABS specimens. This increased porosity is a cascade effect stemming from CF-ABS filament porosity coupled with poor fiber–matrix interfaces contributing to differing flow of the chopped fiber and polymer matrix and resulting in inner-bead porosity during filament extrusion [33]. The investigation of alternate extrusion methods, processing parameters, and optimization of the processing and treatment of the fiber-reinforced composite filament could provide a solution to mitigate porosity formation within these FFF components. Alternative designs, such as hierarchical honeycomb structures, could provide additional avenues for study. Although tested in a different orientation, Mansour et al. [15] reported ultimate strength values in the range of 32–38 MPa, 40–46 MPa, and 41–47 MPA for hierarchical honeycomb structures of ABS, CNT-ABS, and CF-ABS, respectively. These values fall below the ranges of 45–54 MPa, 46–57 MPa, and 47–53 MPa determined for ABS, CNT-ABS, and CF-ABS in the current study, and this can be explained by differences in the hexagonal structures and process parameters between the studies.

**Figure 7:**
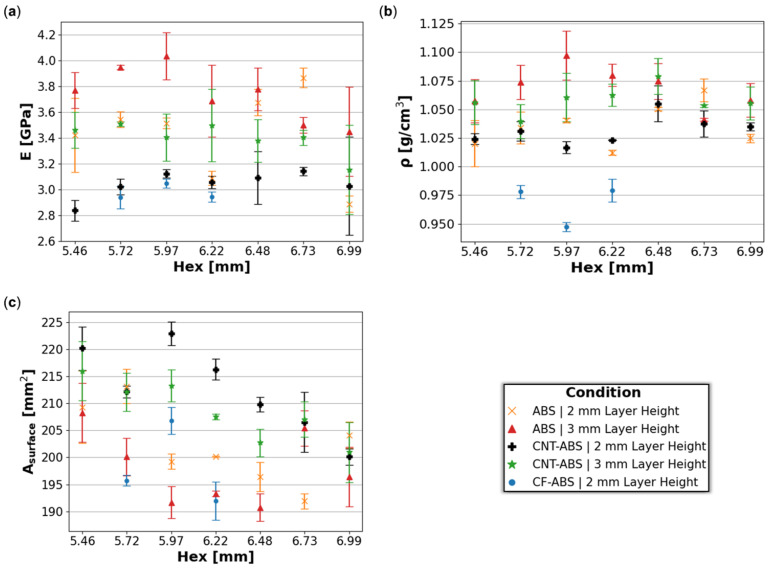
Average (**a**) Young’s modulus (*E*), (**b**) density (ρ), and (**c**) surface area ($A_{surface}$) with standard deviation error bars for ABS, CNT-ABS, and CF-ABS across all hex sizes (5.46 mm, 5.72 mm, 5.97 mm, 6.22 mm, 6.48 mm, 6.73 mm, and 6.99 mm) and print-layer heights (0.2 mm and 0.3 mm).

The average Young’s modulus, *E*, and standard deviation error bars for the ABS, CNT-ABS, and CF-ABS honeycomb coupons, calculated from the linear elastic region of the compressive response, is shown in Figure 7a. Across all hex sizes, from 5.46 mm to 6.99 mm, the Young’s modulus remained relatively constant within each material and print-layer-height combination. The values for CNT-ABS and CF-ABS in this study fall within the ranges of 1.8–4.7 GPa and 1.3–3.6 GPa, respectively, that have been reported in several studies [15,30,34]. However, the Young’s modulus for ABS in the current study exceeded the range (1.6–2.2 GPa) reported by these studies [15,30,34]. This is possibly due to variation in filament properties between batches and vendors in addition to variations between printers and process parameters, yielding printed components with different mechanical properties.

The density, ρ, of each honeycomb material and print-layer-height set generally remained constant across the hex sizes as well, as captured in Figure 7b. Due to the increased propensity for porosity within CF-ABS prints, reduced density in CF-ABS relative to CNT-ABS and ABS specimens was observed. The surface area, $A_{surface}$, measurements in Figure 7c represent the honeycomb cross-sectional area perpendicular to the print direction as calculated by Keyence (Itsaca, IL, USA) analysis software version 3.0.34 for each feature combination. A negative correlation is observed between surface area and hex size for ABS, CNT-ABS, and CF-ABS at 0.2 mm and 0.3 mm print-layer heights. This is expected due to the constrained dimensions of the honeycomb coupons of approximately 25.4 mm for both the length and the width. As a result, increasing the hex size translates to fewer hexes fitting within the 25.4 mm by 25.4 mm area and a decrease in the cross-sectional surface area. This also supports the negative trends for compressive yield and ultimate strengths previously discussed. Although the same printer is used, there are still slight variations in the surface areas between specimens having the same hex size. A combination of processing parameters to include materials, print-layer heights, and fluctuations within the material filament itself impact filament extrusion and the corresponding line width of the extruded filament, providing an explanation for dimensional variance from the input honeycomb design.

**Figure 8:**
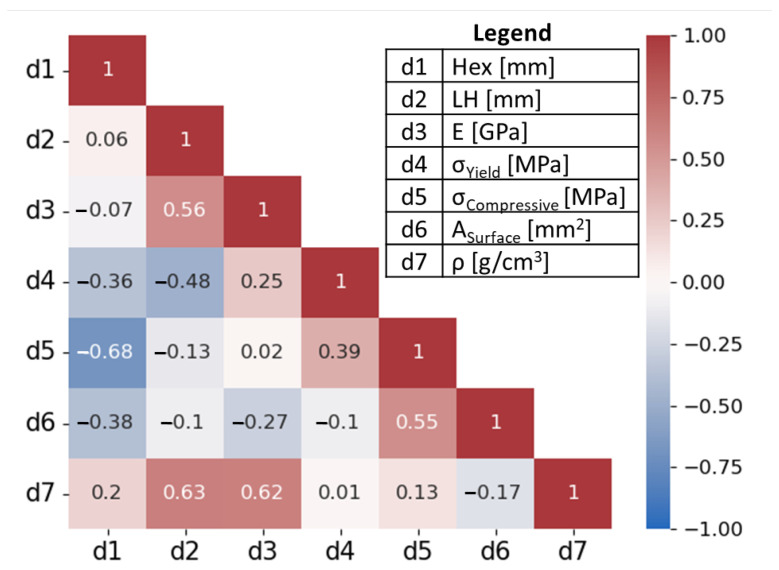
Pearson correlation coefficient matrix heatmap for: hex size (Hex [mm]); layer height (LH [mm]); Young’s modulus (*E* [GPa]); compressive yield strength ($\sigma_{Yield}$ [MPa]); compressive ultimate strength ($\sigma_{Compressive}$ [MPa]); surface area ($A_{surface}$ [mm^2^]); density (ρ [g/cm^3^]). The correlation coefficient value is provided within each grid. Color and intensity correspond to the degree of correlation: red—positive correlation; blue—negative correlation; white—no correlation.

From the 65 data points across all three materials, the Pearson correlation coefficient matrix heatmap between hex size, LH, Young’s modulus, compressive yield strength, ultimate compressive strength, surface area, and density is illustrated in Figure 8. In agreement with observations from Figure 5, Figure 6 and Figure 7, weak-to-moderate negative correlations for [hex size, compressive yield strength], [hex size, compressive ultimate strength], [hex size, surface area], [LH, Young’s modulus], and [surface area, Young’s modulus] are determined. Weak-to-moderate positive correlations for [LH, Young’s modulus], [LH, density], [Young’s modulus, compressive yield strength], [Young’s modulus, density], [compressive yield strength, compressive ultimate strength], and [compressive ultimate strength, surface area] are also observed.

### 2.3. Classification and Regression

**Figure 9:**
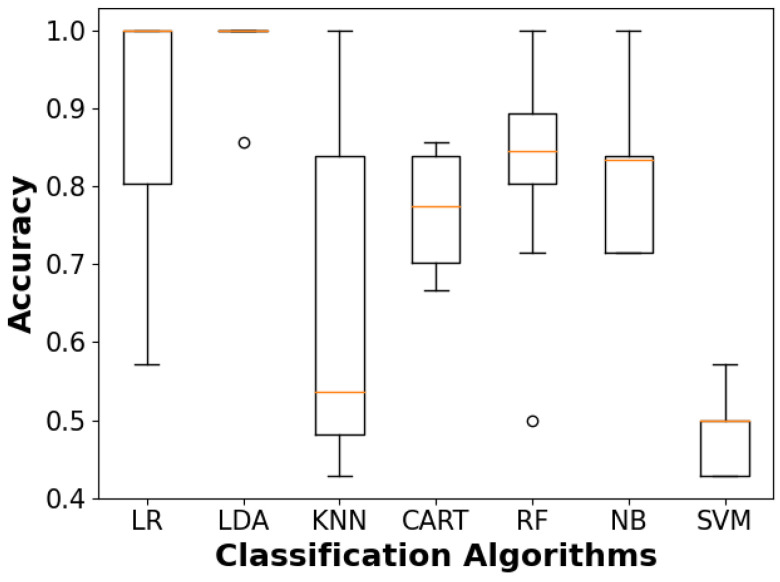
Spot-screening of classification algorithms and corresponding accuracies for material prediction resulting from stratified 8-fold cross-validation with training data: LR—linear regression; LDA—linear discriminant analysis; KNN—k-nearest neighbors; CART—classification and regression decision tree; RF—random forest; NB—naive Bayes; SVM—support vector machine.

A classification model was developed to classify materials by type, and it was trained on a selection of input features with an 80/20% train–test split on the 65 experiment data points. These features include hex size, print-layer height, surface area, mass, volume, density, maximum applied load, yield strength, Young’s modulus, and specific strength. The training accuracies of multiple supervised machine learning classification algorithms following stratified eight-fold cross-validation (CV) are illustrated with the box plot in Figure 9. These algorithms include linear regression (LR), linear discriminant analysis (LDR), K-nearest neighbors (KNN), classification and regression decision tree (CART), random forest (RF), naive Bayes (NB), and support vector machine (SVM). The spread of the accuracy for each algorithm depicts the model’s consistency across each cross-validation split. Here, KNN’s wide spread is indicative of varied performance depending on the cross-validation set, whereas SVM might demonstrate a smaller spread but also has a consistently lower accuracy. Of these algorithms, LDA seems to perform the best, with an average accuracy of 0.98 and a standard deviation of 0.05, as summarized in Table 1. The average accuracies and standard deviations for KNN and SVM are 0.64, 0.21] and [0.48, 0.05], respectively. LR provided the second-best CV accuracy at 0.89 and a standard deviation of 0.16, followed by RF with values of 0.84 and 0.14, respectively. Although the accuracy metric alone does not comprehensively assess model behavior and could be biased toward overfitting, the stratified CV approach helps alleviate these concerns for an initial model down-selection, and these results can subsequently be supported by descriptive metrics, including recall, precision, and F1-score.

**Figure 10:**
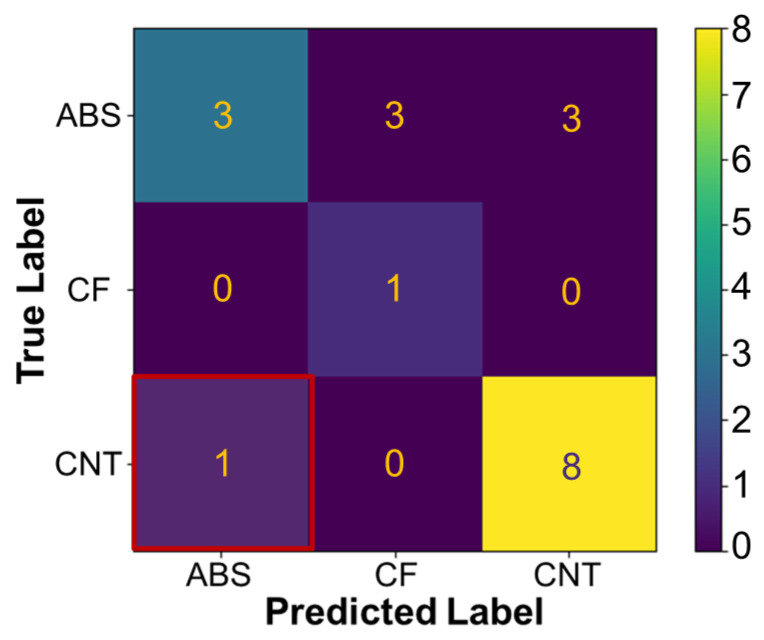
Confusion matrix of linear discriminant analysis predicted labels and true labels based on the test data set: ABS, CF (CF-ABS), and CNT (CNT-ABS).

A down-selection was made based on previously discussed performance, and the LDA model was validated with the unseen test data. A confusion matrix (Figure 10) depicts the model-predicted label of ABS, CF-ABS, or CNT-ABS for a given set of honeycomb features with the true label. Entries in the top-left to bottom-right diagonal correspond to accurate predictions, while counts outside of this diagonal were inaccurately predicted. The overall LDA classification accuracy, taken as the ratio of correct predictions over the total number targets, was found to be 0.92. It was found that the model provided one incorrect prediction of ABS when the material was, in fact, CNT-ABS. This is likely due to overlap in some of the features for each material, such as compressive yield strength, Young’s modulus, and density, and due to the low number of data points available for training. However, it is shown that the LDA classification model performed sufficiently well on the test data, with precision, recall, and F1-score metrics for each material class provided in Table 2. Classification of ABS had a reduced precision and F1-score due to the incorrect label prediction, CNT-ABS had a slight drop to its recall and F1-score, and the F1-score of 1 for CF-ABS demonstrates the model’s well-balanced ability to recall the material class with precision. The caveat is that the small data set resulted in only a single CF-ABS data point remaining in the test set for validation, and a single error resulted in 0.75 precision for ABS classification. Although the model performed reasonably with the current data, by conducting additional experiments and gathering more data, the model could be expanded and refined with more extensive training and testing sets for added utility and improved accuracy. Also not explored is hyperparameter tuning, which could provide an alternate method for improving model performance. However, this step was not deemed necessary based on the current results.

A regression model, taking the form $y=m_{i}X_{i}+\ldots +m_{n}X_{n}+b$, was developed to predict the ultimate compressive strength of honeycomb specimens based on a selection of input features and an 80/20% train–test split of the 65 experiment data points. In this formula, *X* represents the features, m is the feature coefficients, and b is the intercept. These features include material, hex size, print-layer height, surface area, mass, volume, and density. Multiple regression algorithms were trained, and the coefficient of determination, $R^{2}$, to evaluate the fit of model predictions was determined through validation with the test data, with the results shown in Table 3.

**Figure 11:**
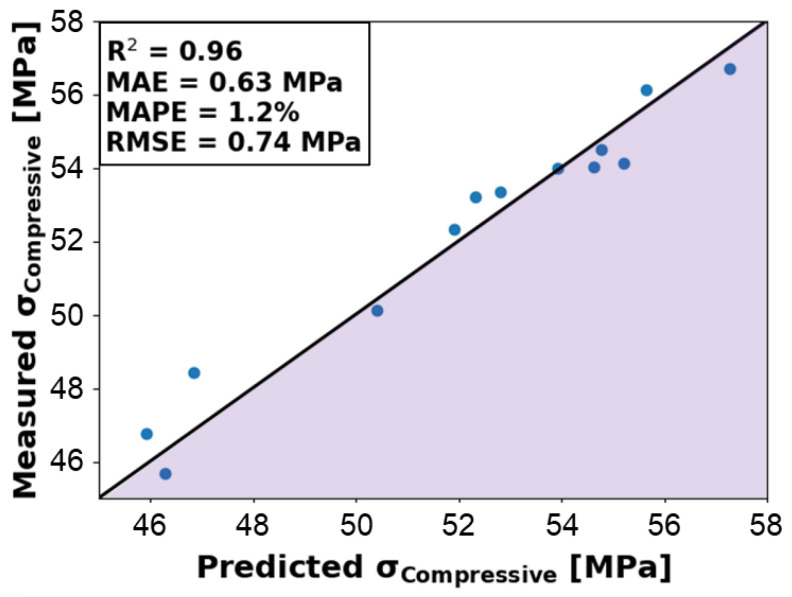
Predicted ultimate compressive strength from trained linear regression model evaluated against experimental measurements for ABS, CNT-ABS, and CF-ABS, resulting in an *R*^2^ of 0.96, mean absolute error (MAE) of 0.63 MPa, mean absolute percentage error (MAPE) of 1.2%, and a root mean square error (RMSE) of 0.74 MPa. Predictions falling on the y = x correspond perfectly with experiments.

The trained algorithms include ridge regressor, linear regression, k-neighbors regressor, gradient boosting regressor, random forest regressor, extra trees regressor, decision tree regressor, and lasso regression. Although *R*^2^ has limitations, such as neglecting prediction accuracy, this metric is still informative for evaluating regression performance [35]. Of these algorithms, lasso regression had the weakest performance, with an $R^{2}$ of 0.36, whereas the top-performing algorithm was linear regression, with an $R^{2}$ of 0.96. Ridge regressor and gradient boosting regressor fell short, with $R^{2}$ values of 0.91 and 0.88, respectively. The mean absolute error (MAE) values of these algorithms also provide insight to model performance and are calculated as the average absolute error between the predicted and the actual ultimate compressive strength. The linear regression MAE of 0.63 MPa corresponds to highly accurate predictions given the range of ultimate compressive strength falls between 46 MPa and 58 MPa. The MAE also tends to decrease with decreasing $R^{2}$ and has a value of 2.34 MPa for the lasso regression algorithm. Based on these findings, the linear regression model predictions evaluated against corresponding experimental measurements are further analyzed in Figure 11. The y = x line demarcates predictions that perfectly correspond to experiment measurements, with under-predicted values falling below the line and over-predicted values above. It is observed that an equal distribution of over- and under-predicted values occurs across the full range of ultimate compressive strength values within the test data set. This signals that the model is not apparently biased toward over- or under-predicting and can perform well across the design space for which it was trained. These observations are supported by the root mean square error (RMSE), 0.74 MPa, and the mean absolute percentage error (MAPE), 1.2%, for the 13 test samples, which represent a good measure of predictive accuracy for the model when compared with experiments. Table 4 summarizes the intercept, features, and corresponding coefficients for this linear regression model.

One-hot encoding was utilized to incorporate categorical features into the previously described linear regression model. In this method, a value of 0 or 1 is assigned to the feature depending on whether it is false or true, respectively. These categorical features included material, hex size, and layer height. An approach with hex size and print-layer height as continuous values will help reduce the number of features and result in a more generalized linear regression model. Table 5 summarizes the intercept, features, and corresponding coefficients for the generalized linear regression model. Here, mass was also dropped as a feature due its redundancy with volume and density resulting in a negligible effect on model performance. This linear regression model demonstrated good predictive accuracy and similar performance to the first model, yielding an *R*^2^, MAE, MAPE, and RMSE of 0.95, 0.61 MPa, 1.2%, and 0.77 MPa, respectively, for the 13 test samples. It should be noted that, similar to the classification model, additional experiments would be beneficial for increasing the training data available and for expanding the utility of the model. However, the linear regression model in its current state performed well at accurately predicting the ultimate compressive strength of the honeycomb specimens in this work.

## 3. Materials and Methods

The specimens in this work were 3D-printed on a Makerbot Replicator 2X (MakerBot Industries, LLC One MetroTech Center, Brooklyn, NY, USA). In addition to material type, print process parameters that were varied included the dimensions of the hexagons comprising the honeycomb structures and the print-layer height, as summarized in Table 6. The 3D-printed filament material, manufactured by 3DXTech, included pure ABS, multi-walled carbon nanotube reinforced ABS, and 5 wt.% chopped carbon fiber reinforced ABS, which will now be referred to as ABS, CNT-ABS, and CF-ABS, respectively. Hex sizes, defined as the distance between opposite walls of a hexagon, were 5.46 mm, 5.72 mm, 5.97 mm, 6.22 mm, 6.48 mm, 6.73 mm, and 6.99 mm. Specimens were printed with individual layer heights of 0.2 mm and 0.3 mm and were printed to overall length, width, and height dimensions of 25.4 mm, 25.4mm, and 12.7 mm, respectively. Each specimen had an open face on the top and bottom layers and maintained a constant wall thickness. Processing features that remained constant across all prints included nozzle diameter, 0.4 mm; filament diameter, 1.75 mm; print speed, 120 mm/s; print temperature, 230 °C; and bed temperature, 110 °C.

**Figure 12:**
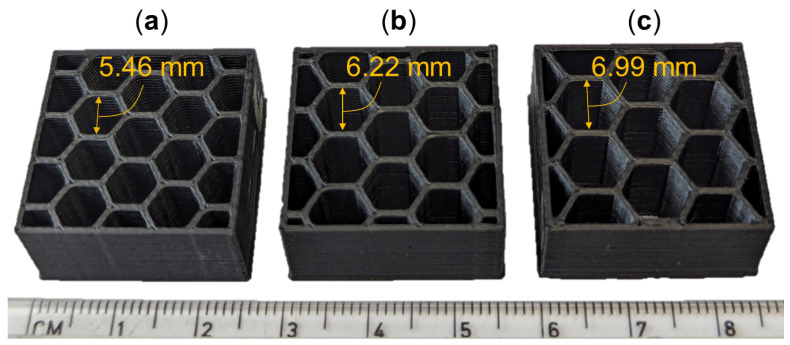
Honeycomb specimens with several hex sizes 3D-printed by FFF: (**a**) 5.46 mm hex; (**b**) 6.22 mm hex; (**c**) 6.99 mm hex.

Figure 12 shows a selection of coupons across the range of hex sizes that fabricated for compression testing; they were loaded at a rate of 6 mm/min and nominally conformed to ASTM C365 [36]. Figure 12a–c show hex sizes 5.46 mm, 6.22 mm, and 6.99 mm, respectively. The overall lengths, widths, and heights of the specimens remained constant, resulting in a reduced number of hexes for coupons with increased hex sizes. Several coupons for each hex size and layer height were fabricated with ABS and CNT-ABS materials, while CF-ABS specimens were of ancillary focus and were only printed with a 0.2 mm layer height and hex sizes of 5.72 mm, 5.97 mm, and 6.22 mm.

**Figure 13:**
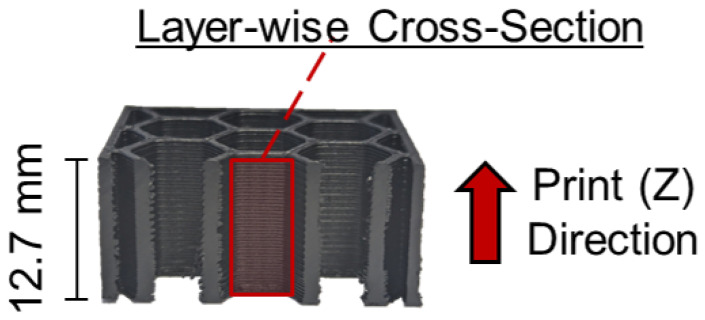
Cross-section of a 3D-printed honeycomb compression coupon in-plane with the print (Z) direction. The region of interest for the optical microscope is highlighted.

A subset of specimens fabricated to the aforementioned specifications were machined to obtain sample cross-sections in-plane with the print direction, as highlighted in Figure 13. These samples were mounted in epoxy and were subsequently polished for examination by optical microscope. It should be noted that Vakharia et al. [7,30] reported pull-out of particulate reinforcement from PLA and ABS composites due to the machining and polishing steps. Additional examination of cross-sections perpendicular to the print direction and surface area measurements were conducted with a VHX-7000N (Keyence, Itsaca, IL, USA) digital microscope. Individual images were stitched together with the microscope’s software (version 3.0.34) to obtain panoramas of the entire cross-section. From these panoramas, the software calculated values for cross-sectional area. Prior to testing, the mass and several measurements each for the length, width, and height by digital caliper (Mitutoyo, Aurora, IL, USA), of all coupons was recorded. A prior investigation by Vakharia and co-authors [30] conducted thermogravimetric analysis, scanning electron microscopy, energy dispersive spectroscopy, and porosity quantification of pure ABS, CNT-ABS, and CF-ABS material systems, and this is not repeated in this work.

**Figure 14:**
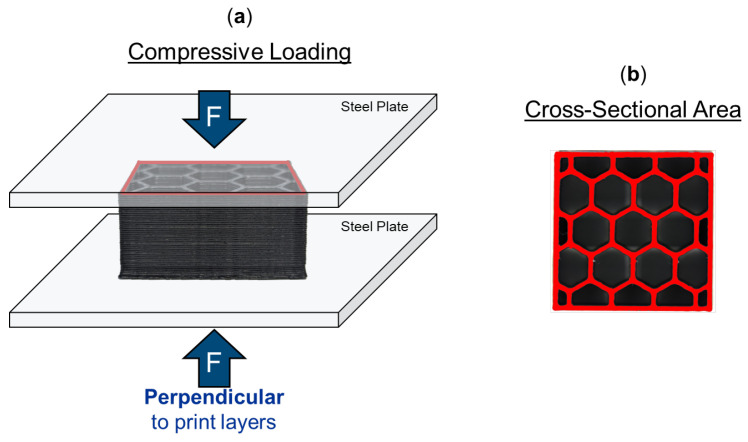
Schematic of honeycomb specimen compression testing: (**a**) honeycomb coupon sandwiched between two steel plates within a test frame, where force is applied perpendicular to the print layers; (**b**) cross-sectional area of honeycomb where load is applied.

For compression testing, coupons were placed in an Instron 8562 test frame, with the open hexagonal cells in contact with flat steel plates on either end, as illustrated in Figure 14a. The load was applied through the honeycomb cross-sectional area (Figure 14b), which had to be determined for each honeycomb due to changing hex sizes. Digital image correlation was conducted with an ARAMIS (Trilion Quality Systems, Plymouth Meeting, PA, USA) system. Two specimens for each ABS and CNT-ABS test condition and three specimens for each CF-ABS test condition were tested. The Young’s modulus, compressive yield strength, and compressive strength were determined from the test data. Although an increased sample size could be beneficial, the obtained data and confidence intervals provided in Figure 5, Figure 6 and Figure 7 are sufficient to discern trends in mechanical properties across materials, hex sizes, and print-layer heights. Supervised machine learning models leveraging publicly available Scikit-learn [37] packages were developed to classify materials and predict the compressive strength based on process parameters and material features. An 80/20% train–test split of the processing features and experimental results was utilized, with the test data reserved for model validation. This split helps alleviate concerns related to missing features from a minimized training set and provides a sufficient test set to reduce potential performance bias. A stratified k-fold cross-validation with eight splits was implemented to ensure an equal proportion of data from each material was included in each split. Following training, the model was validated on the previously unseen test data.

## 4. Conclusions

Several 3D-printed honeycomb specimens of ABS, CNT-ABS, and CF-ABS demonstrated property variations stemming from porosity within the as-supplied filament and due to the influence of processing conditions during fabrication. Examination of honeycomb cross-sections across multiple hex sizes and print-layer heights revealed strong correlation with inter-layer porosity. By increasing the hex size and print-layer height for both CNT-ABS and ABS, a significant increase in porosity was realized within the FFF specimens. Furthermore, the incorporation of carbon reinforcement promoted porosity in terms of both size and quantity, where CNT-ABS specimens contained visibly increased porosity relative to pure ABS specimens. This is, in part, due to treatment with reinforcement material and the resulting adhesive strength with the matrix influencing the quality of extruded filament. These process and structure relationships demonstrated a cascading effect on properties and performance. Increased porosity and weaker inter-layer adhesion resulted in repeated failure of compression coupons in their upper print layers. Although ABS specimens generally reported higher compressive yield strength than carbon-reinforced specimens, CNT-ABS specimens reached higher ultimate compressive strength values due to the compaction of porosity upon yielding and subsequent strengthening. CF-ABS was weaker than ABS and CNT-ABS in terms of both yield and ultimate compressive strengths, having an increased magnitude of porosity, as affirmed by its reduced density.

Based on these findings, it is recommended that these materials are printed with the smallest print-layer height to achieve reduced porosity and improved performance under compression. The raster path and filament extrusion width should be monitored to ensure proper cohesion of adjacent paths, especially at vertices. By leveraging a fully sealed enclosure to attain a uniform elevated temperature prior to printing, the inter-layer adhesion throughout the print and the mechanical properties are expected to improve. Due to the increased propensity for porosity in carbon-reinforced ABS, these materials are more likely to benefit from refinement of processing procedures.

Classification and regression supervised machine learning models were developed from the experimental data by leveraging publicly available tools. Although only a relatively small data set was available for model training and testing, a stratified sampling approach and a broad sweep of available classification and regression algorithms resulted in well-performing models. The linear discriminant analysis material classification model demonstrated a classification accuracy of 0.92: only predicting one label incorrectly. The precision, recall, and F1-score metrics also supported the capability of this model to predict the ABS, CNT-ABS, and CF-ABS material classes based on process parameters and mechanical properties. For predicting the ultimate compressive strength, the linear regression model proved to be unbiased and accurate, with an $R^{2}$ of 0.96 and its mean absolute error of 0.63 falling well within an acceptable margin for values in the range of 46 MPa to 58 MPa. By conducting additional testing, the performance and potential utility of these models to assist with informing and guiding carbon-reinforced honeycomb design for novel 3D-printed structures can be expanded.

## Figures and Tables

**Table 1:** Training results from spot-screening multiple machine learning algorithms for material classification. Stratified 8-fold cross-validation (CV) average accuracies and standard deviations are provided.

Algorithm	CV—Accuracy	Std. Deviation
Linear Regression	0.89	0.16
Linear Discriminant Analysis	0.98	0.05
K-Nearest Neighbors	0.64	0.21
Decision Tree	0.81	0.10
Random Forest	0.84	0.14
Naive Bayes	0.81	0.09
Support Vector Machine	0.48	0.05

**Table 2:** Test results for material classification with linear discriminant analysis. Precision, recall, and F1-score statistical metrics are provided.

Material	Precision	Recall	F1-Score
ABS	0.75	1.00	0.86
CF-ABS	1.00	1.00	1.00
CNT-ABS	1.00	0.89	0.94

**Table 3:** Results from screening multiple regression algorithms for the prediction of ABS, CNT-ABS, and CF-ABS compressive strength. The *R*^2^ and mean absolute error for each algorithm is provided.

Algorithm	*R* ^2^	Mean Absolute Error [MPa]
Ridge Regressor	0.91	0.83
Linear Regression	0.96	0.63
K-Neighbors Regressor	0.70	1.70
Gradient Boosting Regressor	0.88	0.99
Random Forest Regressor	0.79	1.40
Extra Trees Regressor	0.82	1.28
Decision Tree Regressor	0.79	1.5
Lasso Regression	0.36	2.34

**Table 4:** The intercept, features, and corresponding coefficients for a linear regression model with one-hot encoding for material, hex size, and print-layer height: b—intercept; SA—surface area; V—volume; D—density; M—mass; H1 (hex 1)—5.46 mm; H2—5.71 mm; H3—5.97 mm; H4—6.22 mm; H5—6.48 mm; H6—6.73 mm; H7—6.99 mm; LH1 (layer height 1)—0.2 mm; LH2—0.3 mm.

b	SA	V	D	M	ABS	CF-ABS	CNT-ABS	H1	H2	H3	H4	H5	H6	H7	LH1	LH2
−20.0	−0.107	19.9	59.7	−7.20	−1.25	0.0150	1.23	2.06	2.21	1.40	−0.256	−0.105	−1.7	−3.62	0.963	−0.963

**Table 5:** The intercept, features, and corresponding coefficients for the generalized linear regression model with continuous values for hex size and print-layer height and one-hot encoding for material: b—intercept; SA—surface area; V—volume; D—density.

b	SA	V	D	ABS	CF-ABS	CNT-ABS	Hex Size	Layer Height
−10.0	−0.205	24.5	63.7	−1.97	1.72	0.250	−3.15	−1.69

**Table 6:** Processing features for testing.

Feature	Properties
Material	ABS|CF-ABS|CNT-ABS
Hex Size [mm]	5.46|5.72|5.97|6.22|6.48|6.73|6.99
Layer Height [mm]	0.2|0.3

## Data Availability

The raw data supporting the conclusions of this article will be made available by the authors on request.

## Abbreviations

The following abbreviations are used in this document:

ABS	Acrylonitrile butadiene styrene
AM	Additive manufacturing
CART	Classification and regression decision tree
CNT	Carbon nanotube
CF	5 weight-percent chopped carbon fiber
CV	Cross-validation
DIC	Digital image correlation
FDM	Fused deposition modeling
FFF	Fused filament fabrication
KNN	K-nearest neighbors
LDA	Linear discriminant analysis
LH	Layer height
LR	Linear regression
MAE	Mean absolute error
MAPE	Mean absolute percentage error
NB	Naive Bayes
PLA	Polylactic acid
RF	Random forest
RMSE	Root mean square error
SEM	Scanning electron microscope
SVM	Support vector machine
